# Exceptionally high carbon fixation and nitrogen assimilation rates in microbial mats of an alkaline soda lake

**DOI:** 10.1093/ismejo/wraf226

**Published:** 2025-10-09

**Authors:** Yihua Liu, Alyse K Kiesser, Agasteswar Vadlamani, Angela Kouris, Marc Strous

**Affiliations:** Department of Earth, Energy, and Environment, University of Calgary, 750 Campus Drive NW, Calgary, AB T2N 1N4, Canada; Department of Microbiome Science, Max Planck Institute for Biology, Max-Planck-Ring 5, Tübingen, BW 72076, Germany; School of Engineering, University of British Columbia Okanagan, 1137 Alumni Ave, Kelowna, BC V6T 1Z4, Canada; Department of Earth, Energy, and Environment, University of Calgary, 750 Campus Drive NW, Calgary, AB T2N 1N4, Canada; Synergia Biotech, 4220 23 St NE, Calgary, AB T2E 6X7, Canada; Department of Earth, Energy, and Environment, University of Calgary, 750 Campus Drive NW, Calgary, AB T2N 1N4, Canada; Synergia Biotech, 4220 23 St NE, Calgary, AB T2E 6X7, Canada; Department of Earth, Energy, and Environment, University of Calgary, 750 Campus Drive NW, Calgary, AB T2N 1N4, Canada

**Keywords:** cyanobacteria, primary productivity, nitrogen assimilation, protein-SIP, proteomics, carbon stable isotope probing, nitrogen stable isotope probing, Calis-p, urea

## Abstract

Alkaline soda lakes, characterized by high pH and high concentrations of sodium and dissolved carbonates, support diverse alkaliphilic microbial communities. Using stable isotope probing with ^13^C-bicarbonate, ^15^N-ammonium, ^15^N-nitrate, and ^15^N-urea, we measured assimilation rates for carbon and nitrogen by microbial mats of alkaline Goodenough Lake, Canada. Our results showed extremely high carbon fixation rates averaging 24 g C/m^2^/day, equalling or exceeding rates measured fifty years ago in African alkaline soda lakes. Urea consumption occurred both during the day and during the night, but assimilation mainly occurred during the day. Ammonium assimilation was stable between day and night. Apparently, cyanobacteria preferred urea as a nitrogen source, whereas heterotrophs preferred ammonium. Two different cyanobacteria dominated the microbial mats, *Nodosilinea* and *Sodalinema*. Using Orbitrap mass spectrometry, we only observed assimilation of ^13^C bicarbonate by *Sodalinema*, but not by *Nodosilinea.* The latter might focus on different carbon sources, such as urea. Strong negative correlation between their abundances in proteomes also supported niche partitioning between these two cyanobacteria.

## Introduction

Soda lakes are characterized by high concentrations of dissolved carbonates and sodium, alkalinity, and pH [1–3]. Soda lakes occur worldwide, mainly in arid and semiarid zones in central Asia (e.g. Kulunda Steppe, Russia; Lonar Lake, India), North America (e.g. Mono Lake, US; Cariboo Plateau, Canada), East Africa (Nakuru Lake, Kenya; Manyara Lake, Tanzania) and East Australia [2, 4–9]. Despite being among the most extreme aqueous ecosystems known on earth, soda lakes are a paradise for diverse alkaliphilic (alkalinity-loving) microbial communities [1, 2, 4], responsible for the assimilation and metabolism of carbon (C), nitrogen (N), and sulfur (S) as well as other elements [2, 7]. They are also among the most productive systems known in terms of primary productivity or carbon fixation [1], generally exceeding 4 g C /m^2^/(12 h) and reaching up to 19 g C /m^2^/(12 h) [7, 8, 10–12]. Those numbers are much higher than the global mean for streams and lakes, around 0.6 g C /(m^2^ day) [7, 8, 10], making soda lakes a potential terrestrial CO_2_ sink [13]. Recently, soda lake microbial communities have been the focus of research addressing microbial community structure, elemental (such as C, N, and S) cycling [2, 6, 10, 14–18], and carbon fixation [9, 10, 19–21]. The high carbon fixation potential of soda lake microbial communities has drawn the interest of engineers in the field of carbon capture biotechnology [6, 21, 22]. However, we are unaware of any studies that have quantified the rates of carbon assimilation in soda lakes since the 1970s and 1980s, when the assays were either indirect or involved estimations that impact the accuracy [11, 12, 23].

The roles of individual community members in carbon and nitrogen assimilation also remain unknown. The high carbon fixation rates and dense microbial growth require both adequate carbon and nitrogen supply [1, 8, 10, 24]. The unusually high dissolved bicarbonate concentration provides virtually unlimited inorganic carbon for photosynthesis by alkaliphilic phototrophs, especially green algae and cyanobacteria [7]. However, common nitrogen sources (ammonium, nitrate) needed to support high rates of carbon fixation, often only occur in trace amounts in soda lake water [3, 20]. In the past decades, cyanobacteria have been shown to assimilate urea [25–30]. Some freshwater cyanobacteria even prefer urea over other nitrogen sources [31–33], especially at higher pH [34]. In theory, urea assimilation could also be favorable in alkaline soda lakes, where uptake of bicarbonate by cyanobacteria requires energy in the form of ATP [35]. Uptake of urea is advantageous because it is both a nitrogen and carbon source. Indeed, analysis of genomes of alkaliphilic cyanobacteria showed the presence of urease transporters and urease [20, 28, 36], but so far urea uptake in alkaline soda lakes has not been shown experimentally [20, 37–39].

Goodenough Lake, a typical soda lake on the Cariboo Plateau in BC Canada, has a high amount of dissolved inorganic carbon (DIC) such as CO_2_, HCO_3_^−^, and CO_3_^2−^ with concentrations reaching up to ~750 mm C, but little to no dissolved inorganic nitrogen sources (NH_4_^+^, NO_3_^−^) [20, 24]. Goodenough Lake is known for its benthic microbial mats. Observations of Goodenough Lake mats in 1990s showed a nonstratified mat texture with multiple cyanobacterial morphotypes [40]. The ^13^C/^12^C isotope ratios in mat biomass, water, and sediments of Goodenough Lake support non-C-limited photosynthesis [3]. A recent study compiled genomes and proteomes of Goodenough Lake microbial mats, describing two abundant cyanobacterial populations affiliated with *Nodosilinea* and *Sodalinema* (formerly *Phormidium)*. Based on their proteomes, these may have different carbon fixation dynamics and occupy separate ecological niches [20]. With regard to nitrogen, most of the mat’s cyanobacteria expressed genes for ammonium assimilation and many expressed genes for urea assimilation and nitrogen fixation [20, 28]. Rates of nitrogen fixation were recently reported [41], but rates for the assimilation of urea and bicarbonate are still unknown.

To experimentally validate previous genomics inferences, we measured carbon and nitrogen assimilation rates of Goodenough Lake microbial mats using stable isotope probing with ^13^C-bicarbonate, ^15^N-ammonium, ^15^N-urea, and ^15^N-dinitrogen. Assimilation rates for inorganic carbon were as high or higher than previously recorded [7, 8, 10]. Samples were analyzed using isotope ratio mass spectrometry and proteomics, enabling attribution of the bulk of the potential carbon uptake to *Sodalinema* rather than *Nodosilinea*. In addition to measuring urea consumption, we also quantified the mat’s urea production rate. Urea and ammonium consumption showed opposite diurnal dynamics.

## Materials and Methods

### Collection, handling of intact, and homogenized microbial mats

Intact microbial mats were collected in August 2019 near the southwest shore of Goodenough Lake, BC, Canada (51°19′41.4″N 121°38′52.2″W). An intact section of the microbial mat, overlying sediments (~20 cm depth in total) was transferred with a shovel into 42 l polypropylene plastic sample boxes. The shovel was precleaned in lab and washed in nearby lake water prior to sampling to minimize cross-contamination. To keep mat sections intact during transport, 1 l narrow-mouth plastic bottles with cut-off bottoms were placed over the intact mat, cutting into the sediment and completely containing sections of the mat and underlying sediment. Lake water was used to fill the bottles completely, and bottles were capped to prevent sloshing during transport. Additionally, 4 l lake water was collected from the same location and filtered on-site with grade 2 (8 μm) filter paper into sterile bottles, to remove any grit and visible plankton. All samples were transported to a greenhouse at the University of Calgary with exposure to diffused sunlight. The total time from the collection to the start of the incubations was 47 h.

Homogenized microbial mats were prepared directly after sampling by gently stirring microbial mats (~800 ml) sampled from five sites around the shore of Goodenough Lake in June 2018 with ~5 l lake water in an 8 l opaque bucket. The mixture was transported in the opaque bucket with a closed lid for 50 min to an on-site experimental station, followed by subsampling for on-site experiments and storing in a transparent lidless tray for experiments on the following day or night.

The homogenized mat samples were used to estimate potential nitrogen assimilation rates, as homogenization is a standard practice that eliminates structural heterogeneity and facilitates direct comparison across treatments. However, homogenization also removes natural gradients (e.g. of light, oxygen, nutrients) within the mats. Therefore, we used intact microbial mats to estimate actual surficial rates of ^13^C assimilation under preserved structural conditions.

### ^13^C and ^15^N probing of microbial mats

Carbon and nitrogen assimilation in microbial mats were investigated using ^13^C and ^15^N probing respectively. Intact microbial mats were incubated with a filtered Goodenough Lake water spiked with ~2% NaH^13^CO_3_ to trace carbon incorporation over a 48-h period, with samples collected at predetermined intervals. Homogenized microbial mats were incubated separately at non-nitrogen-limiting conditions with one of the four nitrogen supplements containing ^15^N-labelled substrates at a concentration of 0.5 mm during both day and night conditions. The four supplements were urea, ammonium, ammonium with 0.5 mm nonlabelled nitrate, and nitrate with 0.5 mm nonlabelled ammonium. Samples were collected at various time points up to 8 h to monitor nitrogen uptake. All collected samples were preserved appropriately and stored at −80°C for subsequent analysis. Detailed methods are in the supplementary methods under the sections ^13^C probing of intact microbial mats and ^15^N probing of homogenized microbial mats**.**

### Bulk biomass ^15^N/^14^N and ^13^C/^12^C by continuous flow-elemental analysis-isotope ratio mass spectrometry

**Figure 1:**
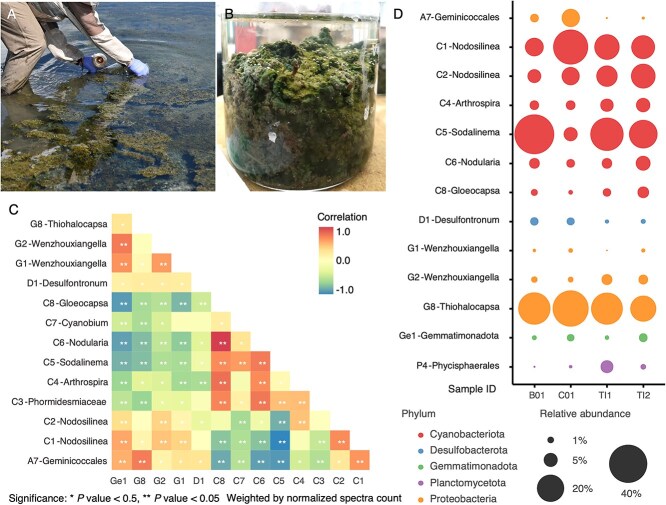
(A) Benthic and floating dark green microbial mats in Goodenough Lake. Note the abundant brine flies lying on the water surface and larvae and exuviae in mats. (B) Intact Goodenough mats generated gas bubbles during incubation at a University of Calgary greenhouse. (C) Relative proteome abundance of the most abundant populations, each associated with a metagenome-assembled genome (MAG), in sample B01 at the start of the incubation, C01 (^13^C labelled), and two mat samples processed directly after collection (TI1 and TI2). The area of the bubbles corresponds to the relative abundance of a population calculated from the spectra counts of peptides unique for each MAG, and normalized to a total of 1 within the sample. Only MAGs with abundance ≥0.001 in all four samples are shown (see also Supplementary Table 2). (D) A heatmap of the correlations between the protein-abundance of each pair of MAGs across all analyzed samples. Only MAGs present in all samples are shown.

The bulk organic ^15^N/^14^N and ^13^C/^12^C ratios of mat biomass were measured using continuous flow-elemental analysis-isotope ratio mass spectrometry (CF-EA-IRMS). Biomass replicates from the same time points were thawed on ice and vortexed at 3000 rpm until thoroughly mixed, centrifuged at 20 × g at 6°C for 20 min with supernatant removed. Biomass pellets were washed with 2 ml 14 g/1000 ml NaCl (similar to the salinity of Goodenough Lake) three times to remove remaining ^13^C bicarbonate, while avoiding lysis of haloalkaliphilic cyanobacteria, as previously described [42]. Thus, because no acid wash was used, the presence of small amounts of inorganic carbonates in the mainly organic microbial mats (Fig. 1A and B; Supplementary Fig. 1) may have slightly diluted the assimilated ^13^C-label during IRMS, potentially leading to the underestimation of the carbon dioxide assimilation rate. Precipitation of (^13^C)bicarbonate during the experiment was unlikely based on previous experiments [42]. Concentrated mat biomass was broken up into smaller chunks in the washing solution by vortexing for 10 s, followed by centrifugation at 6°C, 2000 × g for 20 min. Samples were frozen at −80°C and freeze-dried for 48 h. Lyophilized mats were ground into a fine powder and mixed thoroughly, then portioned for CF-EA-IRMS measurement.

The organic δ^15^N, δ^13^C, nitrogen weight percentage (N_wt_%) and carbon weight percentage (C_wt_%) of these samples were determined on a Thermo DeltaV+ mass spectrometer, following sample combustion in an interfaced Elementar Isotope CUBE elemental analyzer. Briefly, an autosampler dropped samples in tin capsules into a quartz combustion column within the elemental analyzer, where the temperature of the column was maintained at ~1000°C. Dropped samples were “flash-combusted” in the column with an O_2_ pulse injected precisely at the time of each drop. Combustion product gases were swept by Helium carrier flow into a ~650°C reduction furnace, where NO_x_ gases were reduced to N_2_ gas, and further separated into N_2_ and CO_2_ via gas chromatography (GC). Sample gas flow and a reference gas flow spread into the mass spectrometer through a Conflo-IV open split. δ^15^N and δ^13^C were determined by comparing peak areas of the sample to the reference gas [43–46]. ^15^N/^14^N and ^13^C/^12^C were calculated from δ^15^N and δ^13^C standards with ^15^N/^14^N = 0.0036765 (atmospheric nitrogen reference) and ^13^C/^12^C = 0.0111802 (Vienna Peedee Belemnite (VPDB) reference), using the formular described previously [47].

### Quantifying the concentration of dissolved ^15^N-urea by GC–MS

The ^15^N% of dissolved urea in liquid samples was measured by Gas Chromatography–Mass Spectrometry (GC–MS), adopting a previously reported protocol and using 0.05 μmol ^14^N-urea as internal calibrants [48]. Briefly, urea was derivatized in two steps by 1,1,3,3-Tetramethoxypropane (MBS, Sigma) and N-methyl-N-(trimethylsilyl)trifluoroacetamide (MSTFA, Sigma). O-trimethylsily1-2-hydroxypyrimidine, the final derivative of urea, showed a GC peak at m/z = 153, 154, or 155, corresponding to ^14,14^N urea, ^14,15^N-urea, or ^15,15^N urea, respectively. The ^15^N% was calculated as follows.


$$ {}^{15}N\%=\frac{{\mathrm{S}}_{\mathrm{m}/\mathrm{z}155}}{{\mathrm{S}}_{\mathrm{m}/\mathrm{z}153\ \mathrm{peak}}+{\mathrm{S}}_{\mathrm{m}/\mathrm{z}155\ \mathrm{peak}}}a+b $$

with:

S: the area under the corresponding peak,

a and b: the slope and intersect, respectively, of the calibration curve acquired by linear regression of known standards.

Urea-^15^N_2_ (98 atom% ^15^N, 99%, Sigma-Aldrich) and Urea (molecular biology grade, Sigma-Aldrich) were used to generate calibration curves. Mimicked 2018 Goodenough Lake water (Supplementary Table 9), was used to prepare calibration solutions.

^15^N-urea and urea were dissolved and serially diluted separately with mimicked lake water to prepare 0.5 mm solutions. Aliquots of ^15^N-urea and urea were mixed to create 200 μl standards with the following ^14^N/^15^N ratios: 0:1, 1:9, 1:4, and 1:0 in 1.5 ml microcentrifuge tubes as external calibrants. Each ratio was prepared as two technical replicates.

0.05 μmol urea internal calibrant was spiked into each 200 μl of liquid samples in 1.5 ml microcentrifuge tubes. Time zero samples with known amount of ^15^N-urea and urea introduced were used together with the external calibrants for a calibration curve. Samples and standards were freeze-dried overnight in a freeze drier (Labconco Freezone). 240 μl of anhydrous ethanol was added to lyophilized solids, followed by 20 s of vortexing at 3000 rpm and 10 min shaking at 750 rpm at room temperature on a ThermoMixer (Eppendorf). Mixtures were centrifuged at 15 × g for 10 min. Two 100 μl aliquots of supernatant were transferred to two individual 250 μl deactivated glass insert vials (Agilent) in 2 ml screw-top vials (Agilent) and sealed with a 12 mm screw cap (Agilent). 10 μl 0.6 M MBS and 40 μl hydrochloric acid (36.5%–38% solution) were added to each vial, reacted for 1 h and vacuum-dried using a SpeedVac Concentrator (Thermo Scientific) for 2 h at room temperature. Dry residues were sealed and stored in a 4°C fridge. Before running GC–MS analysis, 10 ml of dichloromethane and 20 ml MSTFA were added to each vial, followed by a 2-h incubation at 60°C.

Both standards and samples were randomly arranged in an autosampler linked to the GC system, with a control (no urea) inserted between every five samples. 1 μl liquid was taken by the autosampler from each sample and separated in an HP-5MS (5% Phenyl Methyl) column in the GC (Agilent 6890 N) with Helium as the carrier gas. The injector was kept at 300°C and set to Pulsed Splitless at 12.8 psi (SI: 88256 N/m^2^), 1 min pulse time and 30 ml/min purge flow. The column was kept at 70°C and, after injection, ramped to 150°C at 10°C/min, then to 325°C at 15°C/min. The Mass Selective Detector (Agilent 5973 MSD) was operated in Selected Ion Monitoring (SIM) mode measuring m/z of 153.05, 155.05, 168.05, and 170.05, with the dwell time at 3 s each. The solvent delay (the amount of time the detector was turned off since injection) was set at 4 min, the MS source was set at 230°C, and the MS quad was set at 150°C.

The concentration of ^15^N-urea was calculated by the GC–MS determined ^15^N-urea/^14^N-urea times the concentration of the introduced urea calibrant.

The rate of urea consumption was calculated from the ^15^N-urea concentration in the medium over the course of the experiment as follows.


$$ \mathrm{Urea}\ \mathrm{bioconsumption}\ \mathrm{rate}=\frac{\left({C}_{\mathrm{urea},b}-{C}_{\mathrm{urea},a}\right)\times V}{m\times{\mathrm{Ratio}}_{\mathrm{dry}/\mathrm{wet}}\times \left(b-a\right)} $$

With:

C_urea,b_: the concentration of urea at time point b (h), mmol/l.

C_urea,a_: the concentration of urea at time point a (h), mmol/l.

V: volume of surrounding media, ml.

m: weight of wet biomass.

Ratio_dry_wet_: Assumed to be 0.1, based on a 1:10 ratio of dry mat to wet mat weight.

b – a: time interval (h).

The impact of dilution of ^14^N-urea enriched lake water used to balance the pressure during the sampling was evaluated and found negligible compared to the biological activities.

### Quantifying the concentration of dissolved urea in Goodenough Lake by GC–MS

The concentration of dissolved urea in Goodenough Lake was determined as the concentration of dissolved ^14^N-urea from time zero samples of the ^15^N-urea enriched incubation experiment, ignoring the natural presence of the ^15^N-urea in the lake. The concentration of ^14^N-urea was calculated from the ^14^N-urea% and the average concentration of the ^15^N-urea in the same sample acquired above. The ^14^N-urea% were acquired similarly as the ^15^N-urea% describe above using GC–MS, except not spiking ^14^N-urea internal calibrants to the samples and using a calibration curve with external calibrants made with the following urea to ^15^N-urea ratio: 1:1, 1:9, 1:100, 1:500, 1:1000, and 1:5000.

### SIP/proteomics

Protein was extracted from mat samples, analyzed in a nano liquid chromatography – tandem mass spectroscopy (LC–MS/MS) system, with peptides identified and quantified with Proteome Discoverer software (Thermo Fisher Scientific, version 2.2.0.388). The protein isotope composition was determined with Calis-p 3.0.12 (https://github.com/kinestetika/Calisp).

Proteins were extracted, sequenced and identified as described previously [20]. Briefly, proteins were extracted by a modified filter-aided sample preparation (FASP) protocol [49, 50]. Cells were lysed by bead beating and heating in DDT SDS-lysis buffer [0.1 M concentrations of DDT and SDS], followed by washing with UA solution [8 M urea in 0.1 M Tris/HCl pH 8.5] through 10 kDa filter. The resulting proteins were trypsin digested for 12 h.

Extracted peptides were separated by an RSLC-nano Liquid Chromatograph (Thermo Fisher Scientific, Waltham, MA), and subsequently analyzed on a QExatcive Plus hybrid quadrupole Orbitrap mass spectrometer (Thermo Fisher Scientific). Quadruplicate technical replicates were run as described previously [20]. Identification and quantification of detected proteins were carried out in Proteome Discover version 2.2.0.388 (Thermo Fisher Scientific) using the Sequest HT node. False discovery rates (FDR) were estimated at the peptide and protein level with Percolator Node and FidoCT, respectively and proteins and peptides with FDR >5% were discarded from further analysis. Proteins without protein-unique peptides were discarded.

The database used for peptide identification and construction of metagenome-assembled genomes (MAGs) representing the microbial populations in microbial mats in the Cariboo plateau soda lakes was described previously [20]. In total, 19 513 064 MS/MS spectra were acquired, yielding 3 846 358 peptide spectral matches, and 207 768 identified proteins*.*

The normalized spectral abundance factor (NSAF) representing protein relative abundance was determined by normalizing the number of protein spectrum matches (#PSMs) of a protein by the number of amino acids (#AAs) of that protein to a sample. And the MAG proteome abundance was determined by summing the NSAF for all proteins attributed to a given MAG (identical proteins found in multiple MAGs were not used). The mass spectrometry proteomics data have been deposited to the ProteomeXchange Consortium via the PRIDE [51] partner repository with the dataset identifier PXD059992.

The ^13^C/^12^C and ^15^N/^14^N values of detected peptides were determined with Calis-p 3.0.12, using scored peptide spectra match (PSM) tables and raw MS data (in mzML format) as input. The isotope ratios of MAGs were determined as the medium isotope ratio of the associated peptides passing Calis-p filters. The isotope ratio of each sample was determined as the weighted average isotope ratio of all its MAGs and the unbinned proteins, where the weight was the MS summed intensity of peptides belonging to that MAG or the unbinned peptides together.

The peptide ^13^C/^12^C and ^15^N/^14^N distributions for a given MAG were processed within tidytable 0.11.0 in R 4.3.3. The count of a given peptide at a particular ^13^C/^12^C or ^15^N/^14^N value was calculated as the remainder of the MS summed intensity at that particular value divided by 100 000.

The peptide distribution statistics of lower quantile (q1), median (q2), and upper quantile (q3) peptide ^13^C/^12^C value of a MAG were calculated by Hmisc 5.2-0 in R 4.3.3 using Calis-p detected patterns (Supplementary Data 1). The significance of differences between ^13^C/^12^C composition of a species in two different samples was tested using the exact quantile test, as implemented in EnvStats 3.1.0. R-package, with the distribution of ^13^C/^12^C ratios of peptide MS patterns associated with that species as the input. To reduce the noise caused by the natural heterogeneity of microbial mats, we only considered those peptides present in all samples. Because many samples were compared, we applied the Benjamin-Hochberg correction to the probabilities estimated by the exact quantile test. *P* < .05 were considered significant.

### Calculation of nitrogen assimilation rate and gross mat productivity

The nitrogen assimilation rate and carbon fixation rate were calculated from the increase of ^15^N and ^13^C stable isotopes in the biomass. The carbon fixation rate was used as the gross productivity of the mat. Detailed methods are in the supplementary methods under the sections calculation of nitrogen assimilation rate from bulk biomass ^15^N/^14^N in ^15^N-probed homogenized microbial mats and calculation of carbon fixation rate as gross mat productivity.

## Results

### Observations at the lake and incubations of intact microbial mats

Microbial mats sampled in Goodenough Lake in June 2018 and August 2019 mostly had a thin, dark green top layer. A more fragile lighter green section was present underneath, interspersed with thin pink, white, and brown segments. The mat was benthic, covering the entire bottom of the lake and occasionally extending or floating to the top (Fig. 1A; Supplementary Fig. 1A). The mat, especially when floating, trapped large amounts of gas bubbles. Goodenough Lake microbial mats were poorly stratified and very fragile. Many brine flies were flying over the lake, and high numbers of fly larvae were boring into the microbial mat (Fig. 1A and B; Supplementary Fig. 1). Mat biomass adjacent to exuviae (larval shells that remain after the emergence of the fly) usually had lighter green colors (Fig. 1B; Supplementary Fig. 1C).

During incubations of intact mats, we also observed little stratification, generation of gas bubbles from the mat, and many larvae, both live and dead, exuviae, and brine flies (Fig. 1B, Supplementary Fig. 1B and C).

### Protein-based community structure

We used metaproteomics to infer the microbial community structure for each sample, with peptides assigned to existing MAGs, each associated with a different species of bacterium inhabiting Cariboo soda lakes [20]. We also compared community structures of mats sampled immediately after the start of the incubation (T0) and mats processed directly after collection (TI). Relative abundances for the most abundant microbial populations were consistent across samples, whereas abundances of less abundant members varied (Fig. 1D, Supplementary Table 1 and 2). Cyanobacterial proteins made up an average of 69% of all detected proteins in the mat community, with 37% belonging to the Phormidesmiaceae family (MAGs C1-C4, 18% C1 *Nodosilinea*) and 21% belonging to the Geitlerinemaceae family (MAG C5, *Sodalinema*) (Fig. 1C, Supplementary Table 1 and 2). Proteobacteria were the next most abundant, making up an average of 26%, with 23% Gammaproteobacteria (19% G8, affiliated with *Thiohalocapsa*) and 3% Alphaproteobacteria. C1 *Nodosilinea*, C5 *Sodalinema*, and G8 *Thiohalocapsa* were the most abundant species across all samples (Supplementary Fig. 2). The abundance of C1 *Nodosilinea* and C5 *Sodalinema* cyanobacteria was negatively correlated with each other (Fig. 1C, Supplementary Table 3 and 4). This relationship was apparent across most samples and time points, regardless of the treatment (Supplementary Fig. 3). Overall, the microbial community structure remained stable during incubations (Supplementary Fig. 2). Differences between samples were likely caused by the heterogeneity of the mats. These results were broadly consistent with results reported previously [20].

### Bulk gross carbon assimilation of the community

To determine the assimilation of ^13^C bicarbonate into the mat biomass and the incorporation into proteins, we measured the organic ^13^C/^12^C stable isotope ratio in the total mat biomass using IRMS and in extracted proteins using nano liquid chromatography-tandem mass spectroscopy (LC–MS/MS).

**Figure 2:**
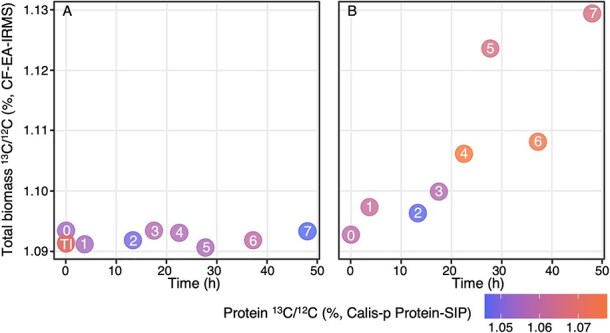
Bulk biomass ^13^C/^12^C during the incubation. The ^13^C/^12^C measurements from IRMS are shown as the y-axis and the peptide measurements are shown using a color scale. The sample time points (1–7, corresponding to 48 h of labelling on the X-axis) are indicated inside the symbols. The biomass ^13^C/^12^C was expected to remain constant in the control (left) and increase with time in the ^13^C labelling experiment (right). Proteomics measurements displayed more noise than IRMS.

IRMS ^13^C/^12^C ratios in control samples (no ^13^C added) were 0.010924 ± 0.001076 (STD), equivalent to δ^13^C of −23.14 ± 0.95‰. During incubations, the ^13^C/^12^C ratio generally increased with time, reaching a maximum of 0.011294 at T7, equivalent to δ^13^C of 10.2‰, indicating the assimilation of ^13^C labelled bicarbonate into the biomass (Fig. 2, Supplementary Table 5). Further, the approximate daytime gross productivity of the mat averaged 24 g C/m^2^/day, with a maximum of 33 g C/m^2^/day and a standard deviation of 5.4 (Supplementary Table 5).

Estimation of ^13^C/^12^C ratios in peptides is less sensitive and precise than IRMS [52, 53]. Extracted protein ^13^C/^12^C values of samples without ^13^C added presented a broader range, 0.010576 ± 0.008216, equivalent to a δ^13^C of −54.06 ± 7.35‰; and ^13^C/^12^C of incubated samples increased above the range of natural abundances for T4, T5, T6, and T7 up to a maximum of 0.01074. Overall, IRMS and peptide ^13^C/^12^C estimates agreed with each other, but peptide estimates had slightly higher ^13^C/^12^C ratios, likely caused by a systematic bias of the mass spectrometer [52] (Fig. 2, Supplementary Table 5). Comparison of the later time points (T4, T5, T6, and T7) to the control experiments indicated carbon assimilation into protein and suggested later sampling times had significant increases in ^13^C/^12^C, even though earlier time points (<T4) showed less ^13^C uptake.

In addition to the bulk ^13^C/^12^C ratios, we used protein-SIP to follow the ^13^C/^12^C ratios of the cyanobacteria C1 and C5 affiliated with *Nodosilinea* and *Sodalinema* in comparison with the most abundant heterotroph G8 (affiliated with *Thiohalocapsa*) during the incubations to determine their contributions to the bulk gross productivity.

**Figure 3:**
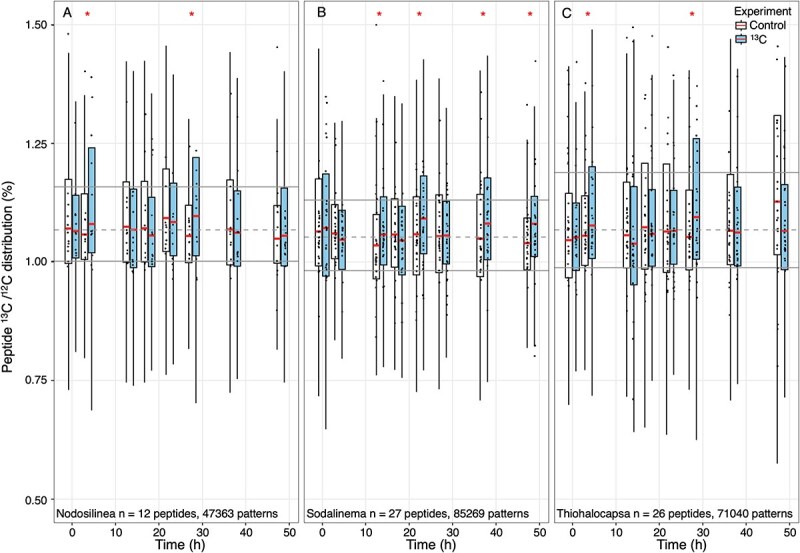
Evidence of ^13^C-labelled bicarbonate uptake by three species of bacteria: (A) C1-Nodosilinea; (B) C5-Sodalinema, and (C) G8-Thiohalocapsa. Boxplots show the median (bold/red line), upper and lower quartile (box), and extension down to the smallest observation or q1–1.5*(q3-q1), whichever is greater, or up to the greatest observation or q3 + 1.5*(q3-q1), whichever is smaller (whiskers) of the ^13^C/ ^12^C content of peptides associated with a species. The total # of isotopic patterns analyzed for each species is shown as n at the bottom of the panels. To reduce noise associated with heterogeneous microbial mat samples, only peptides detected in all samples were considered. The median ^13^C/ ^12^C content of each peptide is shown as a dot, and the # of peptides analyzed for each species is shown as n at the bottom of the panels. For each time point, controls (from unlabelled incubations) are shown in white and treatments (incubations with ^13^C-bicarbonate) are shown in blue. * on top (red) shows significantly higher ^13^C/ ^12^C in treatments than controls based on Benjamin Hochman adjusted *P* < .05 obtained using the exact quantile test. Significant differences may result from the assimilation of ^13^C-bicarbonate or from biological heterogeneity.

Overall, C5, *Sodalinema*, median ^13^C/^12^C increased above the control median ^13^C/^12^C for time points T3 to T7 (Fig. 3B), with significant differences observed in T2, T4, T6, and T7. The ^13^C/^12^C in C5 increased with incubation time, agreeing with the trend in the bulk IRMS measurements (Fig. 2 and 3). In contrast, median ^13^C/^12^C was not significantly higher in treatments than controls for C1 *Nodosilinea*, except for two samples (Fig. 3A), suggesting a smaller contribution to the bulk assimilation. Although two significantly high samples were also observed in heterotroph G8, *Thiohalocapsa*, which was unable to assimilate ^13^C bicarbonate (Fig. 3C) [20], three were significantly lower than the control (not shown), suggesting a higher random effect in the heterotroph, likely from the heterogeneity, which was also shown by the varying abundance of each bin in samples (Fig. 1, Supplementary Fig. 2 and 3).

### Assimilation of nitrogen sources during day and night

**Figure 4:**
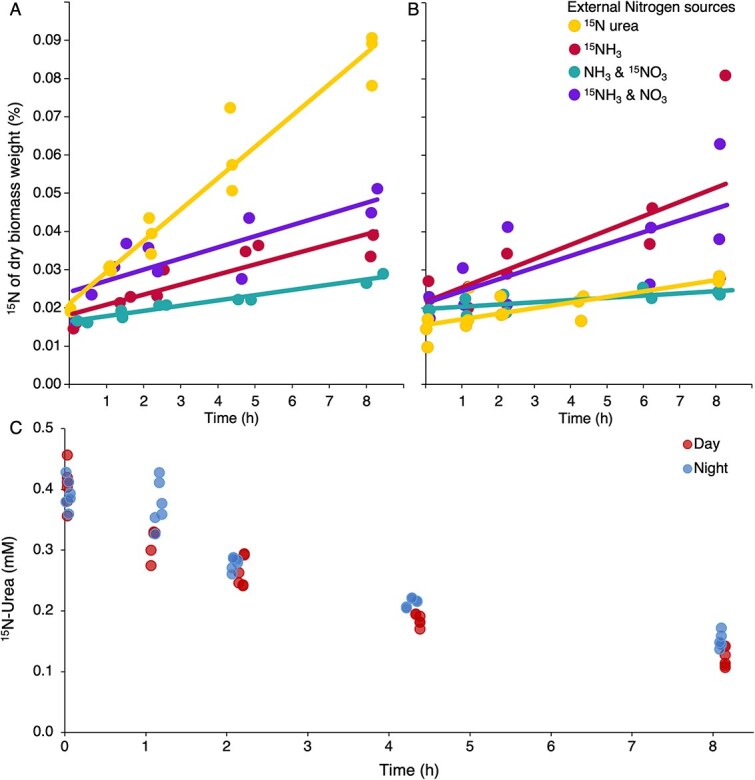
(A and B) ^15^N-nitrogen assimilation by Goodenough Lake mats incubated during day (A) and night (B) with ^15^N-ammonium, ^15^N-urea, ^15^N-ammonium in the presence of unlabelled nitrate, and ^15^N-nitrate in the presence of unlabelled ammonium. (C) The concentration of dissolved ^15^N-urea in the ^15^N-urea incubation experiment during day and night.

Potential nitrogen assimilation rates by homogenized mats in μmol/g/h were calculated from bulk IRMS ^15^N/^14^N ratios of all biological replicates across incubations (Fig. 4A and B, Supplementary Table 6).

The average daytime potential assimilation rate of urea-N was 5.5 μmol/g/h, approximately three times higher than the ammonium assimilation rate, 1.8 μmol/g/h and two times higher than the ammonium assimilation rate in the presence of nitrate, 2.5 μmol/g/h. It was approximately eight times higher than the assimilation rate of nitrate in the presence of ammonium, 0.91 μmol/g/h (Fig. 4A, Supplementary Table 6).

The nighttime average ammonium assimilation rate, 2.5 μmol/g/h, was slightly higher than during the daytime. It was around two and a half times higher than the nighttime urea-N assimilation rate, 0.98 μmol/g/h, and eight times higher than the average assimilation rate of nitrate in the presence of ammonium, 0.39 μmol/g/h (Fig. 4B, Supplementary Table 6).

We also measured the urea consumption rate and compared it to the urea assimilation rate (Fig. 4C). The highest urea consumption rate was presented in the first 2 h of the incubation, then gradually slowed down with the drop of ^15^N urea concentration (Fig. 4C, Supplementary Table 7), indicating that urea consumption and urea assimilation were not exactly synchronized. The urea consumption rate was calculated using linear regression of the data from the first 2 h and averaged among replicates. The urea consumption rate in μmol of urea-N consumed per gram of dry biomass per hour was similar during the day, 6.5 μmol/g/h, and night, 5.5 μmol/g/h. The total amount of urea consumed was similar to the amount of urea assimilated over the course of the experiment. In Goodenough Lake water, the concentration of dissolved urea was 3.9 μm, with a standard deviation of 0.78 (Supplementary Table 8).

The potential assimilation rate of nitrate in the presence of ammonium was low, indicating that the mat microbes preferred ammonium over nitrate (Fig. 4A and B). The assimilation rate of nitrate in the presence of ammonium was twice as high during the daytime than nighttime.

## Discussion

### High gross productivity driven by cyanobacteria

We calculated the gross carbon fixation rate of Goodenough Lake microbial mat samples from the ^13^C enrichment in whole dried biomass (“bulk”) and whole extracted protein, analyzed respectively by IRMS and protein-SIP. The gross carbon fixation rate represents the gross primary productivity. From dried biomass, we calculated a gross bulk daytime productivity of 24 g C/m^2^/day (12 g C/m^2^/(12 h)), with a maximum of 33 g C/m^2^/day (16.5 g C/m^2^/(12 h)) and a standard deviation of 5.4. This range is higher than most previously reported gross rates from the 1970s and 1980s in the African soda lakes, except Lake Aranguadi (Ethiopia), where a gross productivity of 19 g C/m^2^/(12 h) was estimated [8, 10, 11].

Our results confirm exceptionally high gross productivities in alkaline soda lakes measured more than forty years ago with a different set of (indirect) methods used at that time. The gross productivity was comparable to that of equatorial African lakes that are geochemically similar, though lower in pH and alkalinity, yet display major ecological differences. Goodenough Lake displayed a pH of 10.1–10.7 and alkalinity of 0.2–0.65 mol/L [20, 41], higher than most African lakes with pH ranging from 9.6 to 10.5 and alkalinity ranging from 0.05 to 0.26 mol/L [8, 10, 11, 23]. The high carbonate and bicarbonate alkalinity in both soda lake systems provides unlimited inorganic carbon to microbial growth. Although African soda lakes exhibit relatively stable year-round conditions and planktonic cyanobacteria communities [9, 10, 12], Goodenough Lake experiences strong seasonality, blooming in summer and partially evaporating in autumn [41], and hosts centimetres-thick and dense microbial mats with microstructures that create steep light, oxygen, and nutrient gradients. Such gradients likely promote niche partitioning that enhances nutrient flux to consumers and avoids hindered surface photosynthesis from accumulated cyanobacteria, aligning with measured low net productivity [54].

Early soda lake productivity studies in the 1970s–1980s relied on indirect techniques such as dissolved O_2_ measurements in day/night lake water or in light/dark bottles incubations and ^14^C tracer assays. Those techniques provided valuable foundational insights but involved assumptions (e.g. photosynthetic quotient and constant respiration rates for O_2_ to C unit conversion) and uncertainties (e.g. overestimation in ^14^C assays and inconsistent definitions of “day” in hours) [11, 12]. These limitations highlight the challenge of comparing historical rates directly with modern measurements. Our study uses ^13^C stable isotope probing coupled with mass spectrometry, enabling direct quantification of gross carbon assimilation in intact microbial mats—a capability that was not previously available. Although ^13^C stable isotope probing has been applied to microbial mats in hot springs [55, 56], they have not been used in soda lakes. Furthermore, no studies since the 1990s quantified primary production in soda lake systems, apart from a 2002 report on Mono Lake using data collected in the 1980s suggested a max rate around 0.62 g C/m^2^/day (converted from the originally reported unit in μg C/L/day in a depth profile) from phytoplankton incubation [57, 58]. Although our approach overcomes many earlier constraints, we acknowledge its limitations, including incubation under controlled conditions rather than fully *in situ* and the reduced species sensitivity of protein-SIP in highly heterogeneous mats. Despite the limits, our findings represent quantitative evidence of bicarbonate assimilation at the species level in soda-lake mats and extend the methodological toolkit for studying extremophilic carbon fixation.

Our incubations of intact microbial mats showed carbon fixation by C5 *Sodalinema,* but not by C1 *Nodosilinea*, whose bicarbonate uptake remained below detection. This could reflect conditions favouring C5 during incubations or alternative carbon sources for C1, such as urea or organic substrates. Consistent with this, protein-based abundances of C5 and C1 were strongly negatively correlated (r = −0.80, Fig. 1C, Supplementary Fig. 3, Supplementary Table 4), supporting the idea that these two cyanobacteria occupied complementary ecological niches associated with spatially separate compartments within the microbial mats. Previous work already proposed niche differentiation for these two cyanobacteria based on a much higher expression of the light-harvesting pigment phycoerythrin, the carbon concentrating mechanism and reactive oxygen quenching mechanisms by C5 compared to C1 [20]. The ^13^C/^12^C content of isotopic patterns is always highly variable, and our experiment was no exception, as is apparent from the size of the boxes in Fig. 3. However, the algorithm used to infer ^13^C/^12^C content was previously shown to yield reliable median values for the targeted species, despite high variability [52, 53]. Unfortunately, heterogeneous mat samples in combination with the relatively low ^13^C content of the labelled bicarbonate (2%) made it challenging to observe differences in ^13^C assimilation between the two targeted cyanobacteria. Yet, C5-Sodalinema’s relatively consistent trend of high ^13^C content toward the end of the incubation made this organism the most likely candidate to explain the assimilation of ^13^C observed with gold-standard Isotope Ratio Mass Spectrometry (IRMS). Heterogeneity at the millimetre scale likely drove the variance observed in protein-SIP labelling and reduced sensitivity compared to the homogeneous cyanobacterial consortium enriched from the Cariboo soda lakes mats [59].

### Phototrophs and heterotrophs may assimilate different forms of nitrogen

#### Assimilation of ammonium and nitrate

The assimilation rate of ammonium appeared to be independent of light (Fig. 4A and B). The presence of nitrate did not appear to impact ammonium assimilation. Nitrate was less important as a nitrogen source as the nitrate assimilation rate in the presence of ammonium was relatively low compared to urea and ammonium.

The preference for ammonium over nitrate may result from the nitrogen control mechanism [60, 61], which represses the expression of nitrate assimilation genes when ammonium is present [26, 60, 62, 63]. Assimilating nitrate consumes more energy than ammonium, as nitrate must first be reduced, involving two additional steps (nitrate to nitrite, then to ammonium), catalyzed by assimilatory nitrate reductase [63–66]. Future incubations with nitrate as a sole nitrogen source could compare the assimilation rates of nitrate and ammonium.

Published measurements reported low or undetected dissolved inorganic nitrogen (DIN, sum of NH₃ + NH₄^+^+NO₂^−^ + NO₃^−^) in Goodenough Lake, with a single NH₃ measurement of 11 μm in 2017 [20], and DIN was 1.8–8.4 μm in 2021–2022 [41]. Together, these data indicate a small, seasonally variable DIN pool, but the concentrations do not by themselves constrain the assimilation rates. Instead, they are consistent with rapid nitrogen cycling and high biotic demand in a high-pH system where ammonia speciation and volatilization can further reduce the DIN pool.

#### Urea as a source of both nitrogen and carbon for cyanobacteria

Our findings add new insights into urea assimilation by bacteria in the highly productive soda lake ecosystems. Our findings indicate that phototrophs in Goodenough Lake mats, mainly cyanobacteria [20], are the main consumers of urea, as the urea assimilation rate was approximately five and a half times higher during the day than at night (Fig. 4). The 29% higher ammonium assimilation rate at night suggests cyanobacteria may not be the main consumers of ammonium, as cyanobacteria are mainly active as oxygenic photoautotrophs during the day. This diurnal pattern aligns with recent work showing the presence and expression of genes involved in urea assimilation, urease (ureABCDEFG) and urea transporter (urtABCDE) in C1 *Nodosilinea* and C5 *Sodalinema* [20, 28, 36]. A previous study on Goodenough Lake mat microbial communities showed the expression of proteins involved in urea assimilation by almost all cyanobacteria but rarely by heterotrophic species. However, the high urea-N assimilation rate contradicts the idea that ammonium is preferred over urea for cyanobacteria, and that many cyanobacteria species cannot grow with urea as the sole nitrogen source [26, 61, 67–70].

The preferential assimilation of urea by cyanobacteria has recently been observed in several freshwater cyanobacteria [31–34]. The preference is likely due to energetic advantages in the simultaneous uptake of nitrogen and carbon [31, 34], which was recently shown in a freshwater model strain with ^13^C probing [34]. Urea assimilation generally involves three steps: first, the uptake of urea via diffusion through the cell membrane at high concentration, or active transport by an ABC-type transporter; second, the hydrolysis of urea by urease into one CO_2_ and two NH_4_^+^; and third, the incorporation of NH_4_^+^ and CO_2_ into biomolecules [27, 65]. Although the active transport of urea and the synthesis of urease require energy [31, 65], it results in the import of two NH_4_^+^ and one bicarbonate, compared to importing bicarbonate and ammonium by separate ABC-transporters, which also each require energy [71]. Our study suggests that the urea preference previously observed in freshwater also exists in mat-forming haloalkaliphilic cyanobacteria, indicating that the urea preference might be quite general [20].

Although there was an approximately five-and-half-fold difference in the mat’s urea assimilation rate between day and night, the urea consumption rate was similar (Fig. 4C, Supplementary Table 7). This discrepancy suggests urea assimilation into biomass is dependent on light, but the hydrolysis of urea is light-independent. Previous studies suggested that some freshwater cyanobacteria developed biochemical systems to limit the uptake of urea [28, 67], whereas several display “urea gluttony”, rapidly consuming urea in excess of what can be assimilated by biosynthesis, pointing to a lack of controlling mechanisms [31]. Based on our observations, Goodenough Lake cyanobacteria may also hydrolyze urea at a high rate in the dark, but only actively assimilate urea into biomass during the day, when energy from photosynthesis is available.

Direct comparison of the urea assimilation and consumption rate to the dinitrogen fixation rate highlights that urea can contribute substantially to mat N budgets. Mat-slurry-based ^15^N_2_ assay measured around 0.14 μmol/g/h dry-weight/h-based dinitrogen-N assimilation rate (converted from reported 1674.3 nmol/g/d) [41], whereas our measurements showed a urea-N consumption rate of 5.5–6.5 μmol/g/h and a urea-N assimilation rate of 5.5 μmol/g/h (day) and 0.98 μmol/g/h (night). Both datasets derive from bottle incubations of disrupted mat material (slurries or homogenates) that are subject to the impact of sampling variation; still, the order-of-magnitude difference suggests urea uptake represents a major, and in our incubations, dominant pathway supplying N for biosynthesis. Low biomass δ^15^N signatures in the literature have been used to infer dinitrogen fixation [41], but such isotopic patterns can also reflect the assimilation of urea, which is not subject to heavy isotope enrichment in lake water by volatilization like ammonium.

The low concentration of dissolved urea in lake water, together with the high urea assimilation, indicates that of urea may be actively cycled in the system. Potentially, urea in Goodenough Lake could be produced by abundant fly larvae found in the mats [40], or leached from cow excrement from cattle grazing near the lake. Alternatively, mat microbes may cryptically cycle urea: heterotrophic bacteria involved in the remineralization of cyanobacterial necromass could produce urea, which is then immediately consumed by living cyanobacteria [54]. Future studies are needed to further explore the significance of the different sources of urea.

In conclusion, microbial mats from an alkaline soda lake displayed high carbon uptake rates, consistent with previous estimates of very high gross productivity in alkaline soda lakes. Our work showed that in addition to nitrogen fixation, cyanobacteria prefer urea as a nitrogen source, whereas other organisms appear to focus on ammonium, which may in part be generated from nighttime urea hydrolysis. The negative correlation in the abundance of the two main cyanobacteria reinforces the previous suggestion of two separate ecological niches supporting these organisms, though the exact nature of these niches remains to be determined [20].

## Supplementary Material

Supplementary_materials_20251007_wraf226(1)

Supplementary_Tables_wraf226

Supplementary_data_1_wraf226

## Data Availability

The mass spectrometry proteomics data generated and analyzed during this study, the metagenomic database used for peptide identification and the Calis-p protein SIP outputs have been deposited to the ProteomeXchange Consortium via the PRIDE [51] partner repository with the dataset identifier PXD059992. All custom scripts and codes utilized for downstream data processing and analysis of Calis-p outputs are publicly available on GitHub at https://github.com/yihualiued/GEL-protein-SIP-data-analysis.Supplementary tables and additional supporting information are provided as supplementary material accompanying this manuscript.
