# Supplementary material for: Exceptionally high carbon fixation and nitrogen assimilation rates in microbial mats of an alkaline soda lake
Source: ISME J. 2025 Oct 9;19(1):wraf226. doi: 10.1093/ismejo/wraf226 (PMC12598745; doi:10.1093/ismejo/wraf226)
Supplement: Supplementary_materials_20251007_wraf226(1) [file supplementary_materials_20251007_wraf226(1).pdf]

Supplementary Material

Exceptionally high carbon fixation and nitrogen assimilation rates in microbial mats of an alkaline soda lake

Yihua Liu[1,2*], Alyse K. Kiesser[3], Agasteswar Vadlamani[4], Angela Kouris[1], Marc Strous[1*]

[1]Department of Earth, Energy, and Environment, University of Calgary, Calgary, AB, T2N 1N4, Canada

[2]Department of Microbiome Science, Max Planck Institute for Biology, Tübingen, BW, 72076, Germany

[3]School of Engineering, University of British Columbia Okanagan, Kelowna, BC, V6T 1Z4, Canada

[4]Synergia Biotech, Calgary, AB, T2E 6X7, Canada

Summary of contents:

Supplementary Methods

Supplementary Figures 1-3

Supplementary Tables (in a stand-alone .xlsx file): 1-9

1. NSAF abundance T0&TI MAGs
2. Calisp abundance T0&TI MAGs
3. Abundance all samp MAGs
4. Heatmap correlation
5. 13C_12C IRMS vs Calisp & GP
6. 15N_14N biomass IRMS
7. 15N_Urea bioconsumption
8. Urea lake concentration
9. GEL water mimicry

Supplementary Data 1 (in a stand-alone .csv file)

Supplementary Methods

^{13}C probing of intact microbial mats

1 L of bicarbonate incubation media was prepared with 980 mL filtered lake water and 20 mL 8.5 g/L $NaH^{13}CO_3$ in MiliQ water. The total $^{13}C/(^{13}C+^{12}C)$ in the incubation media was estimated at approximately 2%, taking 1% as natural ^{13}C abundance and assuming 500 mM as a typical total dissolved inorganic carbon (including CO_2, HCO_3^- and CO_3^{2-}) concentration, based on measurements collected in previous years.

Intact microbial mats in cut-off plastic bottles were carefully transferred to 600 mL sterile glass beakers with the same diameter, keeping the mat intact without introducing air bubbles. After four hours settling of disrupted sediments, the supernatant was pipetted out from each beaker. 200 mL of bicarbonate incubation media was added to beakers in separate incubations. 200 mL of filtered lake water was added to a separate beaker as the control. All beakers were uncovered and exposed under indirect sunlight in the greenhouse and arrayed in a tray half-filled with fresh tap water to regulate the temperature.

Three samples of ~1 cm^2 of the mat were collected with sterilized tweezers from random locations within the larger plastic sample box before the experiment began, and labelled “time initial, TI”.

A sample of ~1 cm^2 of the mat was collected with sterilized tweezers from each beaker at 0 h (“T0”), day 1 sunset (“T1”, 3.7 h), day 2 sunrise (“T2”, 13.3 h), day 2 at 11 am (“T3”, 17.5 h), day 2 at 4 pm (“T4”, 22.5 h), day 2 sunset (“T5”, 27.7 h), day 3 sunrise (“T6”, 37.2 h), and 48 h (“T7”). Collected mats

were preserved in 2 mL microcentrifuge tubes and cooled on ice for 30-60 min before being centrifuged at 20 X g for 20 min. Supernatants were collected in separate 2 mL microcentrifuge tubes. Both solid biomass and supernatant were stored at -80°C until analysis.

^{15}N probing of homogenized microbial mats

On-site incubations of Goodenough mats in individually sealed serum bottles were done separately at day and night in four groups with different labelled substrates: urea (0.5 mM ^{15}N-urea), NH_4 (0.5 mM $^{15}NH_4Cl$), NH_4-$^{14}NO_3$ (0.5 mM $^{15}NH_4Cl$ & 0.5 mM KNO_3), and NO_3-$^{14}NH_4$ (0.5 mM $K^{15}NO_3$ & 0.5 mM NH_4Cl). Daytime incubations were exposed to direct sunlight at day temperature; nighttime incubations were kept away from any light at night temperature.

During both day and night incubations, 30 g (wet weight) aliquots of homogenized mats were transferred into three 150 mL serum bottles. Bottles were stoppered under collected lake water to avoid air bubbles.

In incubations with ^{15}N-urea, ^{15}N-ammonium, and ^{15}N-nitrate, each bottle was set with a 60 mL syringe (preset syringe) pre-filled with 59 mL lake water, piercing through rubber stoppers as a reservoir to balance internal pressure during injection or sampling. Subsequently, 0.75 mL of a labelled substrate at 0.1 M concentration was injected into each sealed serum bottle, followed immediately by 30 seconds of handshaking, reaching a final concentration of 0.5 mM. Following substrate addition, serum bottles were submerged in a plastic tray with lake water throughout the incubations to regulate temperature fluctuations. The tray was exposed to the environment and sunlight during the daytime incubation and covered with an opaque box during the nighttime incubation to block out light from the working station.

Sampling

Mat community biomass and surrounding liquid were collected at around 0, 1, 2, 4, and 8 h after adding labelled substrates. 2 mL aliquots of the mat community were extracted from each bottle using an 18 G X 4” long needle and transferred to separate 2 mL microcentrifuge tubes. An equal volume of lake water from the pre-set syringe balanced the pressure. Samples were flash-frozen in dry ice and ethanol, and stored on dry ice. For urea incubations, an additional 2 mL samples were filtered through separate 0.2 µm syringe filters and transferred to 2 mL microcentrifuge tubes to determine the concentration of urea in the liquid portion. Samples were fast-frozen and stored on dry ice in the field for four days before being kept at -80°C in the lab for storage.

Calculation of nitrogen assimilation rate from bulk biomass $^{15}N/^{14}N$ in ^{15}N-probed homogenized microbial mats

The assimilation rates of nitrogen substrates - dinitrogen, urea, ammonium, and nitrate - were determined by tracking the increase in $^{15}N\%$ (relative to sample dry weight) over the course of the incubation. The ^{15}N assimilation rate (R_N) was calculated using the following equation:

$$R_N = \frac{0.0036765 \cdot \left(1 + \frac{\delta^{15\mathrm{N}}}{1000}\right)}{1 + 0.0036765 \cdot \left(1 + \frac{\delta^{15\mathrm{N}}}{1000}\right)} \cdot \frac{N_{wt}\% \cdot k}{15\ \mathrm{g/mol}} \cdot \frac{1000\ \mathrm{mmol/mol}}{1\ \mathrm{g}}$$

With,

$\delta^{15}N$ and $N_{wt}\%$: measured by CF-EA-IRMS as described above.

k: The slope of the linear regression line of ^{15}N percentage (relative to sample dry weight) versus incubation time (in hours), with units of h^{-1}.

The resulting assimilation rate was expressed in mmol/g/h, representing the millimoles of nitrogen assimilated per gram of wet biomass per hour.

To further determine the assimilation rates for specific nitrogen substrates, the calculations accounted for the nitrogen content of each substrate.

Calculation of carbon fixation rate as gross mat productivity

The gross productivity of a sample was calculated as:

$$\mathrm{GP_i} = \frac{\mathrm{IRmat_i} - \mathrm{IRmat_0}}{\mathrm{IRDIC} - \mathrm{IRmat_i}} \times \mathrm{M/d_i}$$

$$(\mathrm{i} = 0,1,2,3,4,5,6,7)$$

With:

GP_i: Gross productivity calculated from Ti sample.

$IRmat_i$: The $^{13}C/^{12}C$ of the bulk Ti sample (calculated based on IRMS) or specified populations from Ti sample (calculated based on proteomics)

IRDIC: The $^{13}C/^{12}C$ of dissolved inorganic carbon, measured as 0.0193.

d_i: days of incubation at Ti

M: Mat carbon content per surface area, calculated as:

$$\mathrm{M} = \frac{\text{Dry Weight}}{\text{Wet Weight}} \times \frac{\text{Mat Thickness} \times \text{Area} \times \text{Wet Mat Density}}{\text{Area}} \\ \times \text{Percentage of Organics in Mats} \times \text{Percentage of C in Organics} \\ \cong 500\,\mathrm{g/m^2}$$

In this calculation, we assumed 1:10 as the ratio between the weight of dry mat and wet mat[1]; 1 cm as the mat thickness; 1 g/cm^3 as the wet mat

density; and 50% as the mass percent C in the mat's organics[2–4].

Supplementary Figures

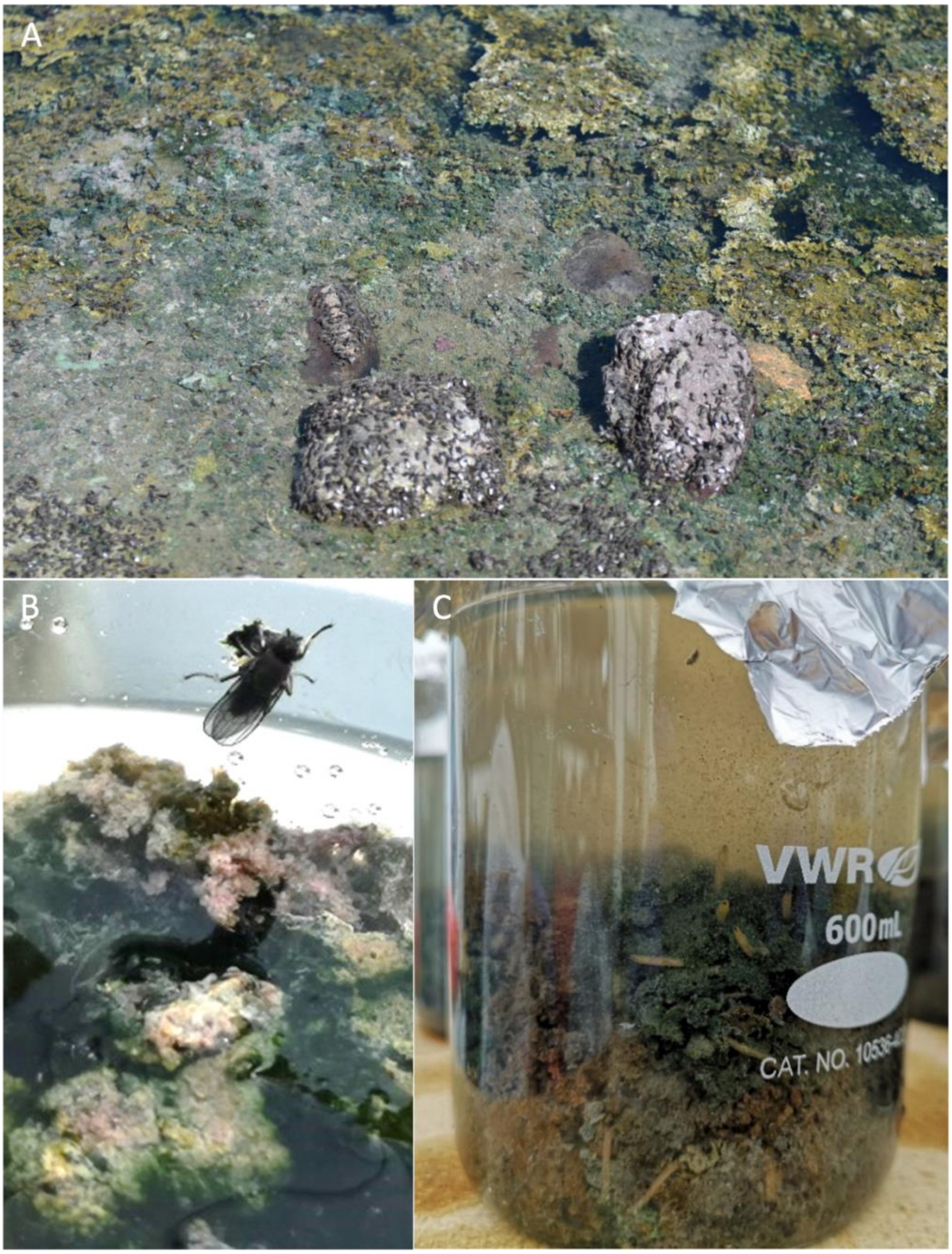

Supplementary Figure 1. Microbial mat morphology in situ and during incubations. Photos of Goodenough mats in situ (**A**) and during incubation (**B** & **C**) at a greenhouse at the University of Calgary, showing the mat textures, larvae activities, and a brine fly.

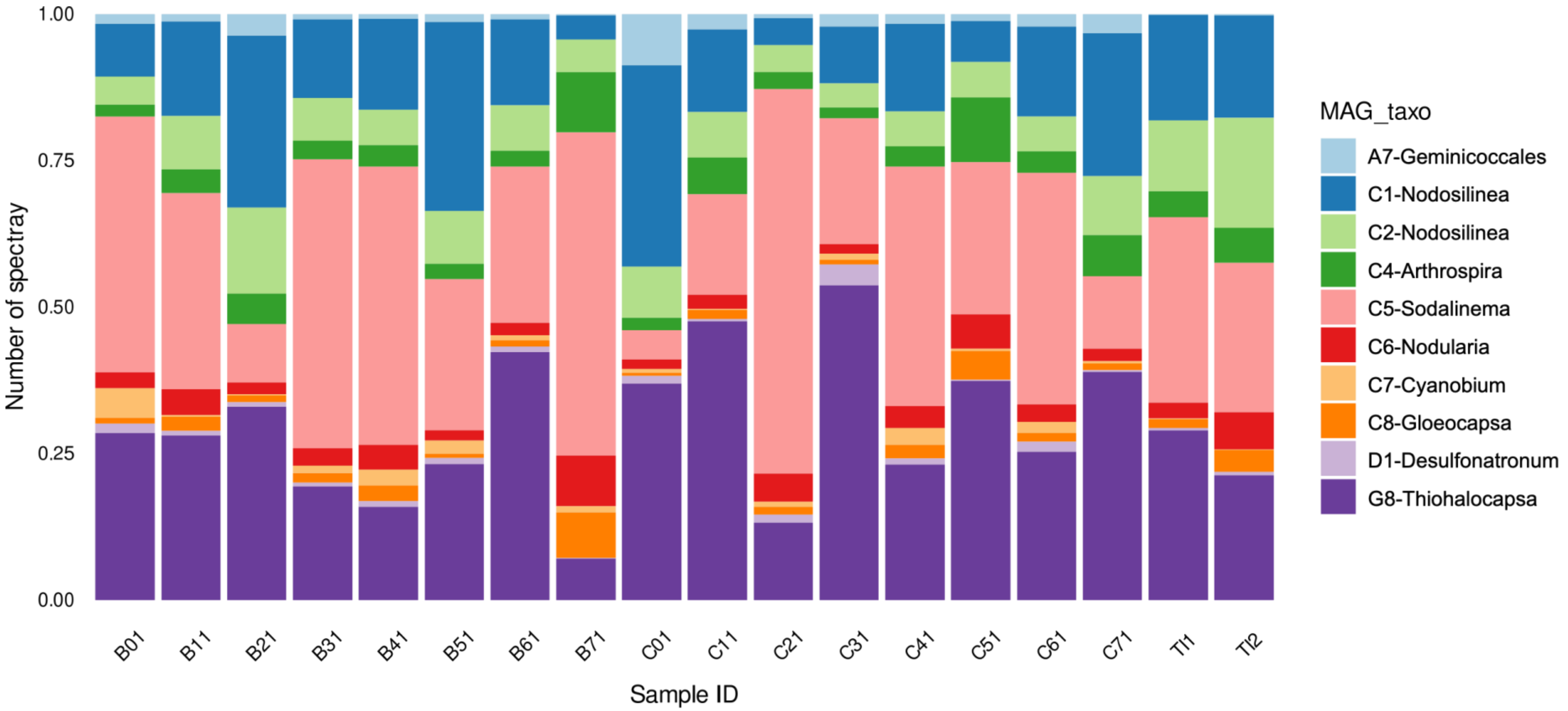

Supplementary Figure 2. Community profile of samples taken during the incubation of intact mats with 13C labelled bicarbonate. Community profiles were based on numbers of peptides associated with metagenome assembled genomes obtained previously [5]

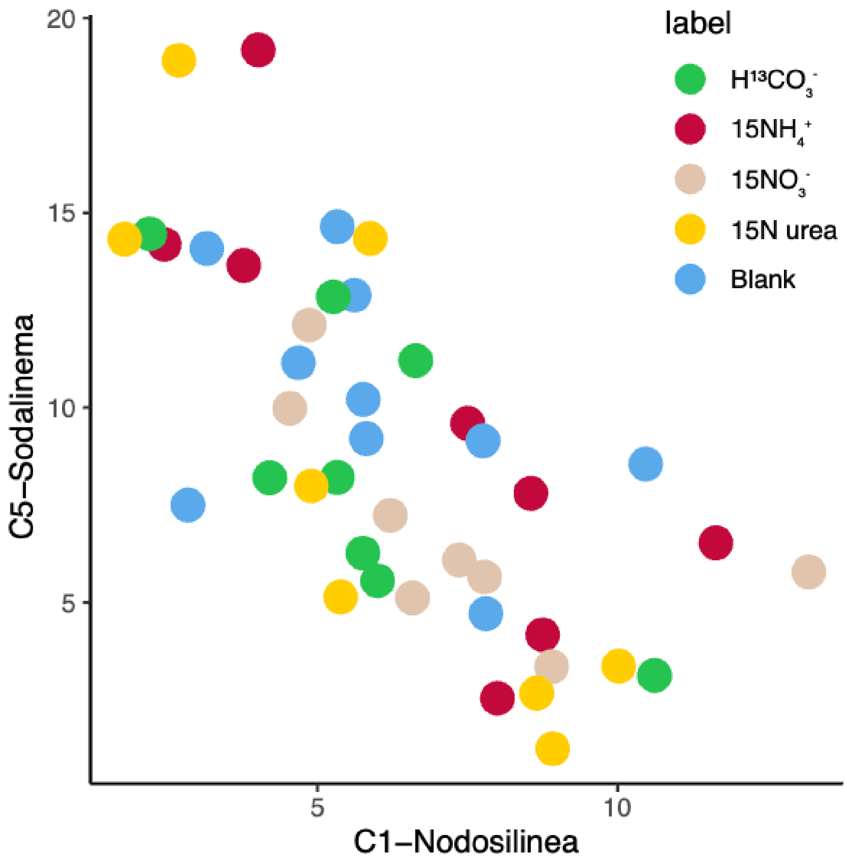

Supplementary Figure 3. Relative abundance based on NSAF values for detected proteins from C1 Nodosilinea and C5 Sodalinema. MAGs in all samples analysed with proteomics. Control experiments (blue), ^{13}C bicarbonate incubations (green), ^{15}N-ammonium (red), ^{15}N-urea (yellow) and ^{15}N-nitrogen (earth).

Bibliography

1. Sharp CE et al. Robust, high-productivity phototrophic carbon capture at high pH and alkalinity using natural microbial communities. *Biotechnol Biofuels* 2017;**10**:84. https://doi.org/10.1186/s13068-017-0769-1

2. Roger PA et al. Chemical composition of cultures and natural samples of N2-fixing blue-green algae from rice fields. *Biol Fertil Soils* 1986;**2**:131–146. https://doi.org/10.1007/BF00257592

3. Ataeian M et al. Direct capture and conversion of CO 2 from air by growing a cyanobacterial consortium at pH up to 11.2. *Biotechnol Bioeng* 2019;**116**:1604–1611. https://doi.org/10.1002/bit.26974

4. Shastri AA, Morgan JA. Flux balance analysis of photoautotrophic metabolism. *Biotechnol Prog* 2005;**21**:1617–1626. https://doi.org/10.1021/BP050246D

5. Zorz JK et al. A shared core microbiome in soda lakes separated by large distances. *Nat Commun* 2019;**10**:4230. https://doi.org/10.1038/s41467-019-12195-5
